# Mechanisms responsible for the synergistic antileukemic interactions between ATR inhibition and cytarabine in acute myeloid leukemia cells

**DOI:** 10.1038/srep41950

**Published:** 2017-02-08

**Authors:** Jun Ma, Xinyu Li, Yongwei Su, Jianyun Zhao, Daniel A. Luedtke, Valeria Epshteyn, Holly Edwards, Guan Wang, Zhihong Wang, Roland Chu, Jeffrey W. Taub, Hai Lin, Yue Wang, Yubin Ge

**Affiliations:** 1 National Engineering Laboratory for AIDS Vaccine, Key Laboratory for Molecular Enzymology and Engineering, the Ministry of Education, School of Life Sciences, Jilin University, Changchun, P. R. China; 2 Department of Pediatrics, Wayne State University School of Medicine, Detroit, MI, USA; 3 Cancer Biology Graduate Program, Wayne State University School of Medicine, Detroit, MI, USA; 4 Department of Oncology, Wayne State University School of Medicine, Detroit, MI, USA; 5 Molecular Therapeutics Program, Barbara Ann Karmanos Cancer Institute, Wayne State University School of Medicine, Detroit, MI, USA; 6 Division of Pediatric Hematology/Oncology, Children’s Hospital of Michigan, Detroit, MI, USA; 7 Department of Hematology and Oncology, The First Hospital of Jilin University, Changchun, P. R. China; 8 Department of Pediatric Hematology and Oncology, The First Hospital of Jilin University, Changchun, P. R. China

## Abstract

Acute myeloid leukemia (AML) continues to be a challenging disease to treat, thus new treatment strategies are needed. In this study, we investigated the antileukemic effects of ATR inhibition alone or combined with cytarabine in AML cells. Treatment with the ATR-selective inhibitor AZ20 caused proliferation inhibition in AML cell lines and primary patient samples. It partially abolished the G2 cell cycle checkpoint and caused DNA replication stress and damage, accompanied by CDK1-independent apoptosis and downregulation of RRM1 and RRM2. AZ20 synergistically enhanced cytarabine-induced proliferation inhibition and apoptosis, abolished cytarabine-induced S and G2/M cell cycle arrest, and cooperated with cytarabine in inducing DNA replication stress and damage in AML cell lines. These key findings were confirmed with another ATR-selective inhibitor AZD6738. Therefore, the cooperative induction of DNA replication stress and damage by ATR inhibition and cytarabine, and the ability of ATR inhibition to abrogate the G2 cell cycle checkpoint both contributed to the synergistic induction of apoptosis and proliferation inhibition in AML cell lines. Synergistic antileukemic interactions between AZ20 and cytarabine were confirmed in primary AML patient samples. Our findings provide insight into the mechanism of action underlying the synergistic antileukemic activity of ATR inhibition in combination with cytarabine in AML.

Cytarabine (ara-C) has been the mainstay induction therapy for most acute myeloid leukemia (AML) patients for the past 40 years^1^. Although many patients respond to induction chemotherapy, the majority of patients relapse leading to overall survival rates of only 25% for adults and 65% for children^2^,^3^. One major mechanism of resistance to chemotherapy is increased DNA damage response (DDR)^4^,^5^. Ataxia–telangiectasia and Rad3 related (ATR) is one of the two chief regulators of the DDR^6^,^7^. It is activated in response to single-stranded DNA structures, which can arise during repair of DNA double-strand breaks or stalled replication forks^7^,^8^,^9^. Most tumor cells have a defective G1 cell-cycle checkpoint and rely heavily on the S and G2 checkpoints for cell survival from DNA damage. Thus, inhibition of ATR may represent a promising means to enhance the antileukemic activities of DNA damaging agents (e.g. cytarabine) in AML cells.

ATR inhibitors have been tested in combination with DNA damaging agents such as gemcitabine, cisplatin, etoposide, carboplatin, oxaliplatin, PARP inhibitors, and ionizing radiation in preclinical solid tumor models, and have demonstrated promising preclinical results^7^,^10^,^11^. Though, an in depth understanding of the mechanism of action when used in such combinations is lacking. ATR plays important roles in multiple cellular functions including cell-cycle arrest, inhibition of replication origin firing, protection of stressed replication forks, and DNA repair^7^. Determining which mechanism contributes in combination regimens will likely deepen our understanding of how ATR inhibitors enhance the antitumor effects of DNA damaging agents and will allow for rationally designed combination therapies for treating AML.

In this study, we investigated the mechanism of action of the ATR-selective inhibitors AZ20 and AZD6738 alone and in combination with cytarabine in preclinical models of AML. We found that AZ20 induced DNA damage and apoptosis, which were independent of CDK1 activity. It also induced DNA replication stress and caused downregulation of ribonucleotide reductase M1 (RRM1) and M2 (RRM2) subunits, which were not dependent on CDK1 activity. The combined treatment with cytarabine and AZ20 or AZD6738 caused increase in chromatin-bound RPA32 and increased γH2AX levels prior to induction of apoptosis, demonstrating that ATR inhibition and cytarabine treatment cooperate to induce DNA replication stress and DNA damage, leading to apoptosis. Our findings provide insight into the mechanism of action underlying the synergistic antileukemic activity of ATR inhibition in combination with cytarabine.

## Results

### ATR inhibition induces proliferation inhibition and apoptosis in AML cell lines and primary patient samples

**Figure 1:**
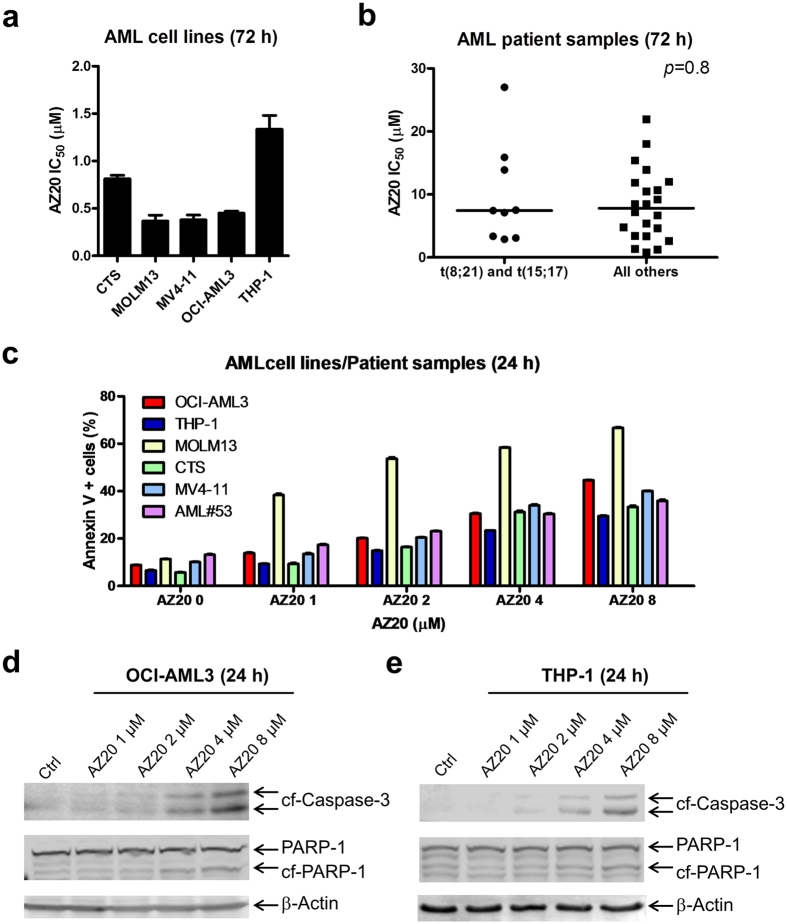
AZ20 induces proliferation inhibition and apoptosis in AML cell lines and primary patient samples. (**a** and **b**) AML cell lines and primary patient samples were treated with variable concentrations of AZ20 in 96-well plates for 72 h and viable cells were determined using MTT reagent. IC_50_ values were calculated as drug concentration necessary to inhibit 50% OD_590_ compared to vehicle control treated cells. AML cell line data are graphed as mean values ± SEM from three independent experiments (panel a). For the patient samples, the IC_50_ values are mean values of duplicates from one experiment due to limited sample. The horizontal lines indicate the median. (**c**) AML cell lines and primary patient sample AML#53 were treated with AZ20 for 24 h and then subjected to annexin V-FITC/PI staining and flow cytometry analyses. Mean percent annexin V + cells ± SEM from one representative experiment performed in triplicates are shown. For cell lines, experiments were repeated three times, while patient sample experiments were performed once due to limited available sample. (**d** and **e**) OCI-AML3 (panel d) and THP-1 (panel e) cells were treated with AZ20 for 24 h. Whole cell lysates were subjected to Western blotting to measure PARP-1 and caspase-3 cleavage. Western blots were repeated at least three times and one representative cropped blot is shown.

To begin our investigation, we used MTT assays to determine AZ20 sensitivities in AML cell lines and primary patient samples. AZ20 IC_50_s were variable, ranging from about 350 nM to 1.4 μM in the AML cell lines (Fig. 1a) and from 800 nM to 27 μM in the primary patient samples (Fig. 1b). The patient samples were separated based on the WHO classification of favorable chromosome abnormalities [t(8;21) and t(15;17); we did not have any inv16 samples to include] and all others [non-t(8;21), -t(15;17), and -inv16]. Based on the samples tested, AZ20 sensitivity appeared to be similar between these two groups (*p* = 0.8, calculated using the Mann-Whitney *U*-test). To assess the effect of AZ20 on AML cell death, we treated AML cell lines and one primary patient sample with 0–8 μM AZ20 for 24 h and subjected the cells to annexin V/propidium iodide (PI) staining and flow cytometry analyses. As shown in Fig. 1c–e, AZ20 treatment induced concentration-dependent apoptosis, as demonstrated by increased annexin V positive cells and increased cleavage of caspase-3 and PARP-1.

### ATR inhibition abrogates the G2 cell cycle checkpoint and induces DNA replication stress, DNA damage, and apoptosis in AML cell lines

**Figure 2:**
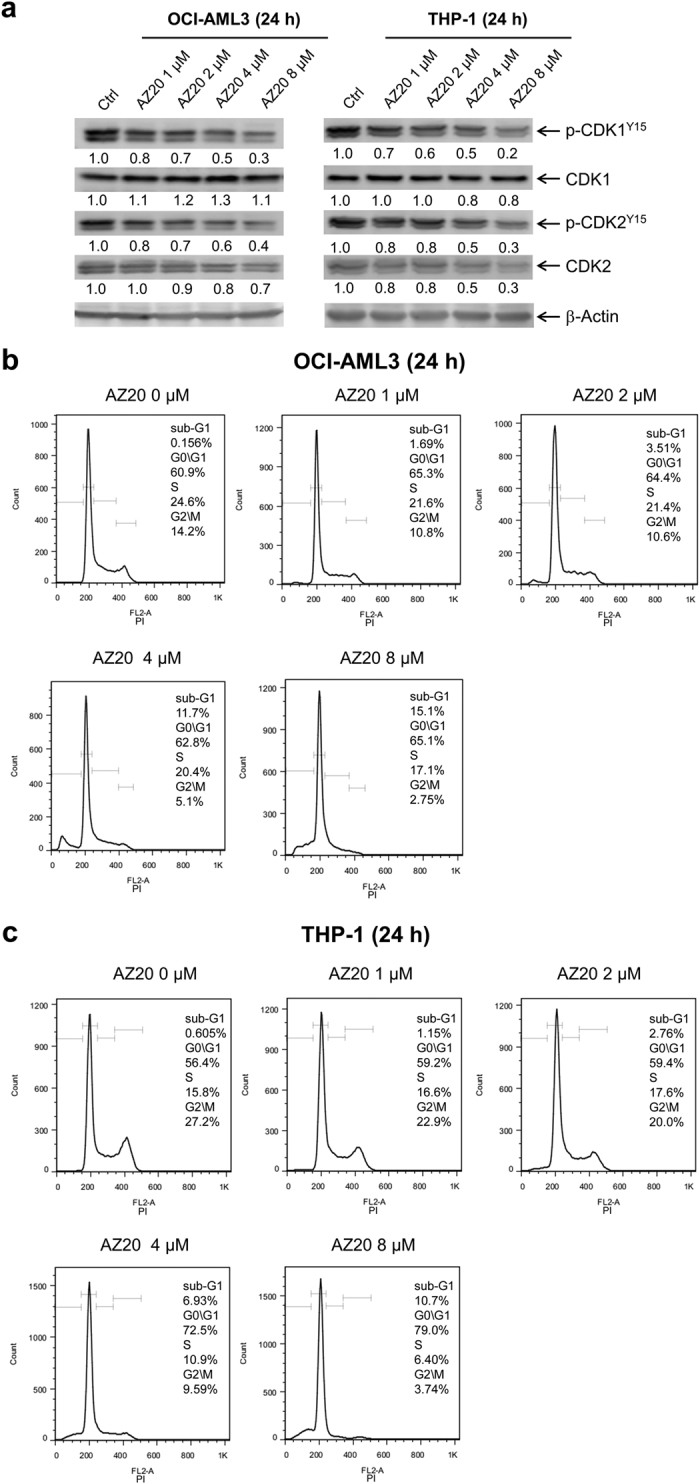
AZ20 abolishes the G2/M cell cycle checkpoint in AML cell lines. (**a**) OCI-AML3 and THP-1 cells were treated with 0–8 μM AZ20 for 24 h. Whole cell lysates were subjected to Western blotting and probed with the indicated antibodies. Densitometry measurements normalized to β-actin and then compared to vehicle control are presented. Western blots were repeated at least three times and one representative cropped blot is shown. (**b** and **c**) OCI-AML3 (panel b) and THP-1 (panel c) cells were treated with 0–8 μM AZ20 for 24 h, then fixed with 80% ice-cold ethanol and stained with PI for cell cycle analysis.

Next, to investigate the effects of ATR inhibition on cell cycle progression, we treated OCI-AML3 and THP-1 cell lines (both are relatively resistant to cytarabine) with AZ20 for 24 h. AZ20 treatment caused concentration-dependent decrease of p-CDK1 (Y15) in both OCI-AML3 and THP-1 cell lines. Although we detected decreased p-CDK2, there was a corresponding decrease in total CDK2 levels, thus the fraction of active CDK2 did not change (Fig. 2a). PI staining and flow cytometry analyses revealed decrease of the G2/M population following AZ20 treatment (Fig. 2b,c). Taken together, these results demonstrate that AZ20 treatment abrogates the G2/M cell cycle checkpoint in THP-1 and OCI-AML3 cells through activation of CDK1.

**Figure 3:**
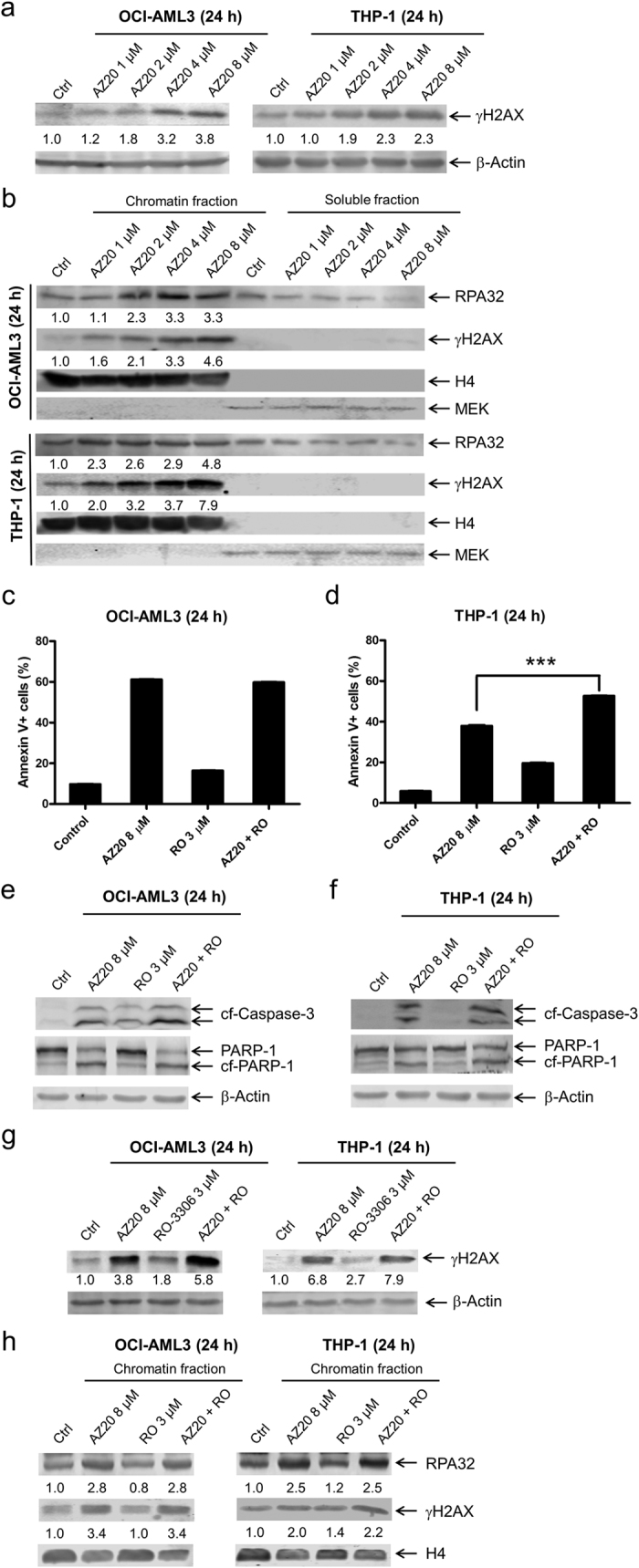
AZ20 induces replication stress and DNA damage in AML cell lines. (**a**) OCI-AML3 and THP-1 cells were treated with AZ20 for 24 h. Whole cell lysates were subjected to Western blotting and probed with anti-γH2AX or -β-actin antibody. Densitometry measurements normalized to β-actin and then compared to control are presented. Western blots were repeated at least three times and one representative cropped blot is shown. (**b**) Levels of RPA32 and γH2AX bound to chromatin and in soluble fractions of AZ20 treated OCI-AML3 or THP-1 cells were analyzed by Western blots. Densitometry measurements normalized to histone H4 and then compared to control are presented. Western blots were repeated at least three times and one representative cropped blot is shown. (**c** and **d**) AML cells were treated with AZ20 in the absence or presence of RO-3306 (RO) for 24 h. Cells were then subjected to annexin V-FITC/PI staining and flow cytometry analyses. Combined treatment was compared to AZ20 treatment alone using pair-wise two-sample t-test. ***Indicates p < 0.001. (**e**–**g**) Whole cell lysates were subjected to Western blot analysis. Densitometry measurements normalized to β-actin and then compared to control are presented. Western blots were repeated at least three times and one representative cropped blot is shown. (**h**) The levels of chromatin-bound RPA32 and γH2AX were analyzed in OCI-AML3 and THP-1 cells after 24 h treatment with AZ20 in the absence or presence of RO-3306. Densitometry measurements normalized to histone H4 and then compared to control are presented. Western blots were repeated at least three times and one representative cropped blot is shown.

To determine if ATR inhibition causes DNA damage, we treated AML cell lines THP-1 and OCI-AML3 with AZ20 for 24 h and then subjected whole cell lysates to Western blotting. AZ20 treatment resulted in a concentration-dependent increase of phosphorylated H2AX (γH2AX), suggesting that AZ20 treatment caused DNA damage (γH2AX is an established biomarker for DNA double-strand breaks^12^, Fig. 3a). Increased chromatin-bound RPA32 and γH2AX were detected after AZ20 treatment (Fig. 3b), reflecting increased DNA replication stress and damage. Next, the AML cells were treated with AZ20 and RO-3306 (a CDK1-selective inhibitor), alone or in combination, for 24 h to determine if CDK1 activation was important for AZ20-induced DNA damage, DNA replication stress, and apoptosis. Treatment with 3 μM RO-3306 for 24 h has been demonstrated to inhibit CDK1 in OCI-AML3 cells leading to G2/M cell cycle arrest and apoptosis^13^. In addition, RO-3306 treatment caused a small increase in apoptosis, demonstrating that this concentration inhibited CDK1 (Fig. 3c,d). In OCI-AML3 cells, it had no effect on AZ20-induced apoptosis, while in THP-1 cells it significantly enhanced AZ20-induced apoptosis (Fig. 3c–f). RO-3306 treatment increased γH2AX levels and slightly enhanced AZ20-induced γH2AX expression in both cell lines (Fig. 3g). RO-3306-induced γH2AX was likely due to the increase in apoptotic cells; γH2AX is a marker of DNA strand breaks, including those generated during late apoptosis^14^. Nonetheless, we did not see a decrease of γH2AX for the combined treatment, indicating that AZ20-induced γH2AX is not CDK1-dependent. RO-3306 treatment, in the absence or presence of AZ20, did not affect chromatin-bound γH2AX or RPA32 (Fig. 3h). Therefore, our data suggests that CDK1 activity does not contribute to AZ20-induced DNA damage and apoptosis. These results indicate that ATR inhibition causes CDK1 activity-independent DNA replication stress, DNA damage, and apoptosis in AML cells.

### Inhibition of ATR results in CDK1-independent downregulation of RRM1 and RRM2

**Figure 4:**
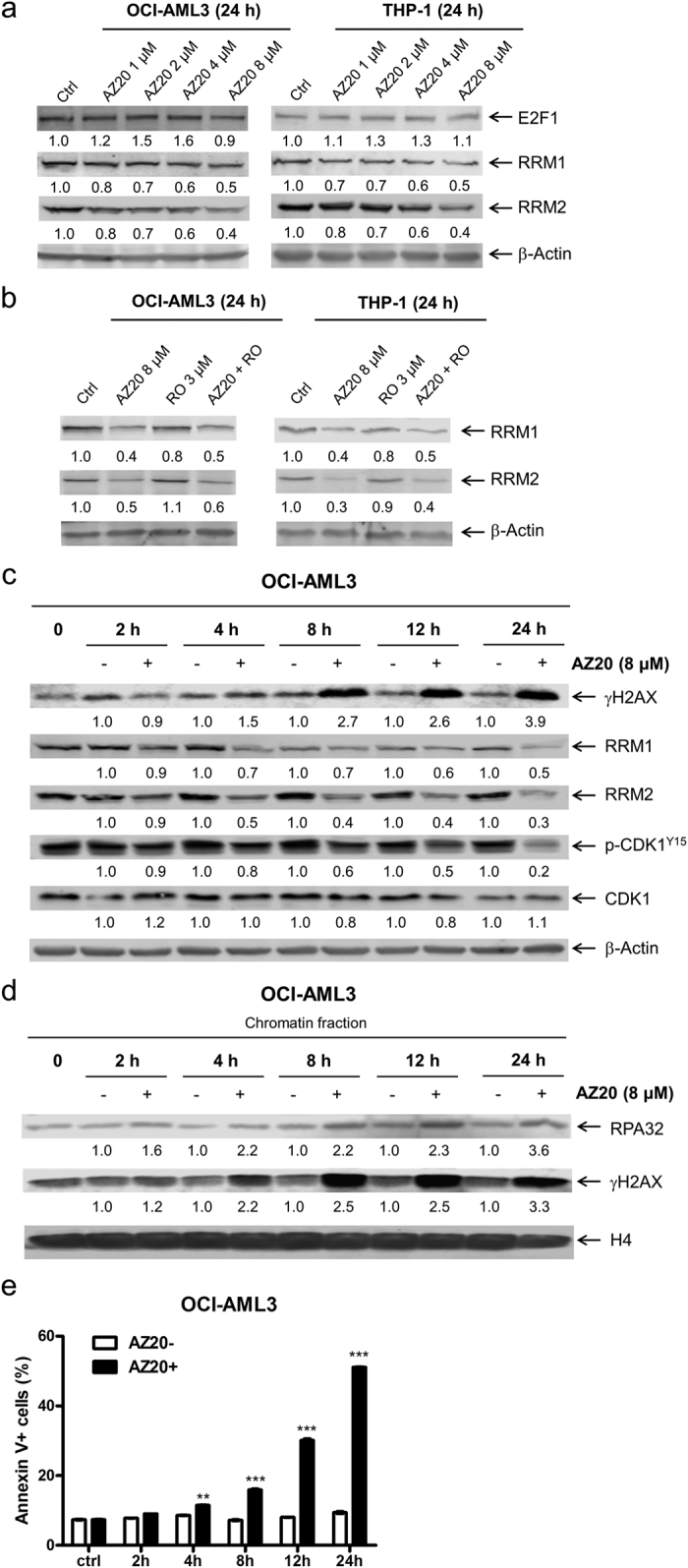
AZ20 treatment causes CDK1-independent downregulation of RRM1 and RRM2 in AML cells. (**a**) OCI-AML3 and THP-1 AML cell lines were treated with variable concentrations of AZ20 for 24 h. Whole cell lysates were subjected to Western blotting and probed with the indicated antibodies. Western blots were repeated at least three times and one representative cropped blot is shown. (**b**) OCI-AML3 and THP-1 cells were treated with 8 μM AZ20 in the absence or presence of RO-3306. Whole cell lysates were subjected to Western blotting and probed with the indicated antibodies. Western blots were repeated at least three times and one representative cropped blot is shown. (**c** and **d**) OCI-AML3 cells were treated with 8 μM AZ20 for 0, 2, 4, 8, 12 or 24 h. Whole cell lysates were subjecte d to Western blotting and probed with the indicated antibodies (panel c). Chromatin-bound RPA32 and γH2AX were analyzed by Western blotting (panel d). Densitometry measurements normalized to β-actin or histone H4 and then compared to control are presented in panels a–d. Western blots were repeated at least three times and one representative cropped blot is shown. (**e**) OCI-AML3 cells were treated with or without 8 μM AZ20 for up to 24 h and then subjected to annexin V-FITC/PI staining and flow cytometry analyses. For each time point, treated and untreated were compared using pair-wise two-sample t-test. **Indicates p < 0.01 and ***Indicates p < 0.001.

It has been reported that ATR promotes RRM2 accumulation via CDK2 and E2F1, limiting DNA replication stress and generation of single-stranded DNA (ssDNA)^15^. Thus, inhibition of ATR may suppress RRM2 expression, leading to DNA replication stress and DNA damage. To investigate this possibility, we treated OCI-AML3 and THP-1 cells with variable concentrations of AZ20 for 24 h and then measured RRM1 and RRM2 expression in the cells. Interestingly, AZ20 treatment caused decreased expression of both RRM1 and RRM2. However, the expression levels of E2F1 remained largely unchanged (Fig. 4a). Further, RO-3306 treatment did not affect RRM1 and RRM2 expression levels (Fig. 4b), indicating that CDK1 activity was not required for the downregulation of RRM1 and RRM2 induced by AZ20 in these cells. These results suggest that AZ20 treatment causes DNA replication stress potentially through downregulation of RRM1 and RRM2.

To determine if DNA replication stress, DNA damage, and downregulation of RRM1 and RRM2 occur prior to induction of apoptosis in response to AZ20 treatment, time course experiments were performed in the OCI-AML3 cells. Our experiments using whole cell lysates revealed a time-dependent increase of γH2AX and decrease of p-CDK1, RRM1, and RRM2 as early as 4 h post AZ20 treatment (Fig. 4c). A similar time-dependent induction of γH2AX and RPA32 was also detected on chromatin (Fig. 4d). Although there was a small (<3% increase compared to vehicle control treatment) yet significant increase in apoptosis at 4 h, a biologically significant increase in apoptosis was not detected until 8 h after AZ20 treatment (Fig. 4e). Taken together, these results suggest that AZ20 treatment causes DNA replication stress and DNA damage prior to induction of apoptosis.

### ATR inhibition synergizes with cytarabine treatment to induce AML cell death and proliferation inhibition

**Figure 5:**
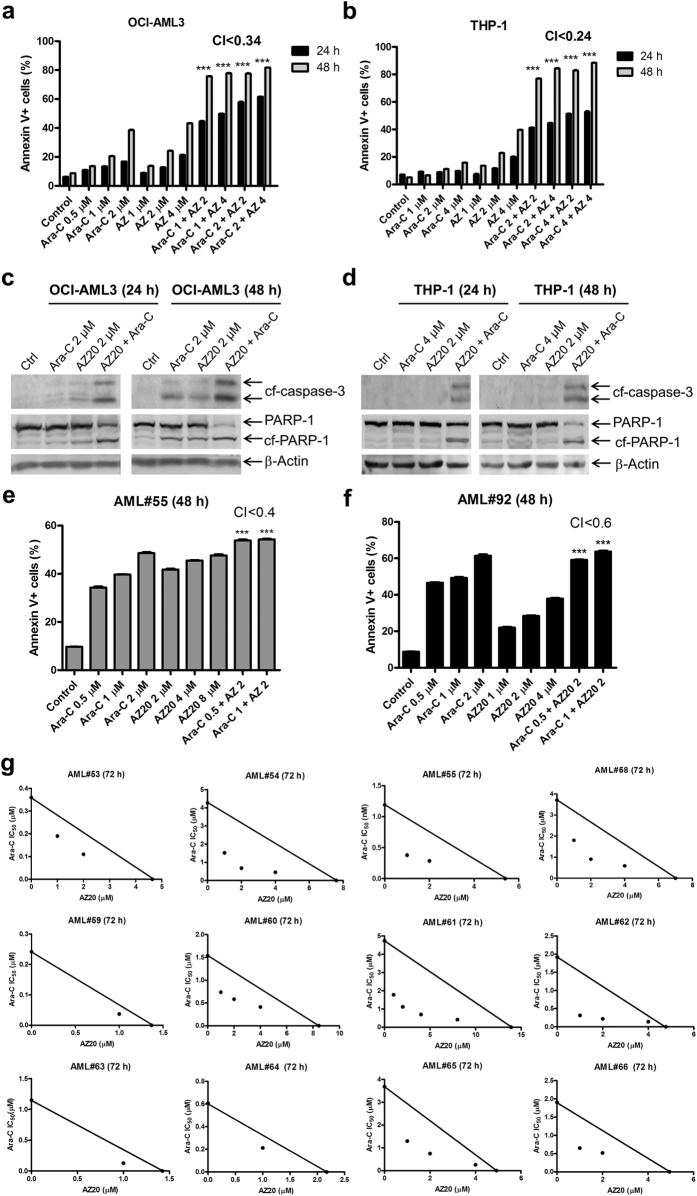
AZ20 synergizes with cytarabine (ara-C) to induce apoptosis and proliferation inhibition in AML cells. (**a** and **b**) OCI-AML3 (panel a) and THP-1 (panel b) cells were treated with cytarabine and AZ20, alone or in combination, for 24 or 48 h and then subjected to annexin V-FITC/PI staining and flow cytometry analyses. CI values were calculated using CompuSyn software. Combined drug treatments were compared to single drug treatment using 1-way ANOVA with Bonferroni post hoc test. ***Indicates p < 0.001. (**c** and **d**) OCI-AML3 (panel c) and THP-1 (panel d) cells were treated with cytarabine and AZ20, alone or in combination, for 24 or 48 h. Whole cell lysates were subjected to Western blotting and probed with the indicated antibodies. Western blots were repeated at least three times and one representative cropped blot is shown. (**e** and **f**) Primary AML patient samples, AML#55 (panel e) and AML#92 (panel f), were treated with cytarabine and AZ20, alone or in combination, for 48 h. Cells were then subjected to annexin V-FITC/PI staining and flow cytometry analyses. CI values were calculated using CompuSyn software. Mean percent annexin V + cells ± SEM from one experiment, due to limited available sample, performed in triplicates are shown. Combined drug treatments were compared to single drug treatment using 1-way ANOVA with Bonferroni post hoc test. ***Indicates p < 0.001. (**g**) Primary AML patient samples were treated with cytarabine and AZ20, alone or in combination, for 72 h and then viable cells were determined using MTT reagent. The IC_50_ values are means of duplicates from one experiment due to limited sample. Standard isobologram analyses of antileukemic interactions were performed to determine the extent and direction of the antileukemic interactions. The IC_50_ values of each drug are plotted on the axes; the solid line represents the additive effect, while the points represent the concentrations of each drug resulting in 50% inhibition of proliferation. Points falling below the line indicate synergism whereas those above the line indicate antagonism.

Next we investigated the effects of AZ20 treatment on cytarabine-induced apoptosis in both AML cell lines and primary patient samples. AZ20 enhanced cytarabine-induced apoptosis, as determined by annexin V/PI staining and flow cytometry analyses, and detection of increased cleavage of PARP-1 and caspase-3 (Fig. 5a–d). The enhancement was synergistic, as indicated by CI (combination index) <0.34. These results were confirmed in 2 primary patient samples (these samples were chosen based on availability of adequate number of cells for the assay, Fig. 5e,f). Additionally, we tested the antileukemic interactions between the two drugs in 11 primary AML patient samples by MTT assays and standard isobologram analyses, which require fewer cells than the apoptosis assay. Interestingly, synergistic antileukemic interactions between the two drugs at the concentrations tested were detected in all the 11 primary AML patient samples (Fig. 5g). To rule-out off-target effects, another ATR-selective inhibitor, AZD6738, was tested in combination with cytarabine. Similar to AZ20, AZD6738 synergized with cytarabine to induce apoptosis in OCI-AML3 cells (CI < 0.06, Fig. S1a,b).

### The combination of AZ20 and cytarabine causes enhanced DNA replication stress, increased DNA damage and apoptosis in AML cells

**Figure 6:**
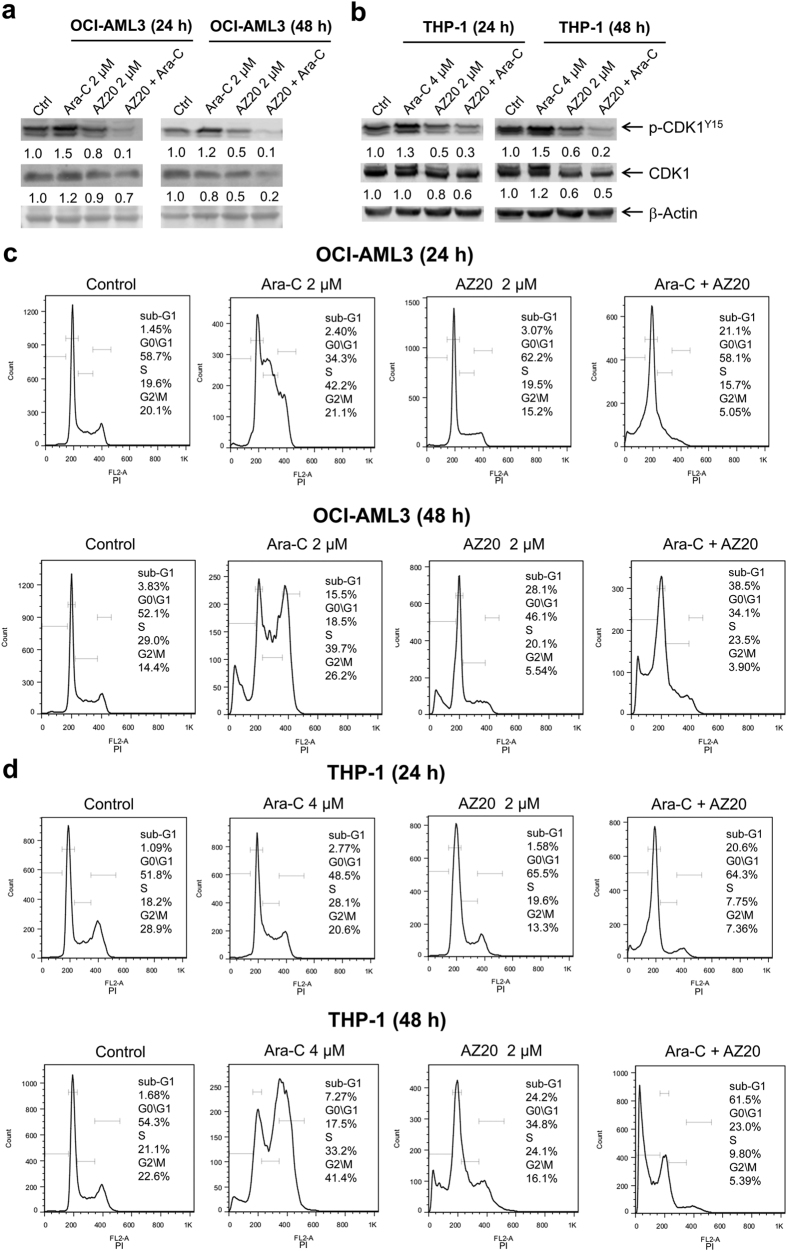
Apoptosis induced by the combined AZ20 and cytarabine (ara-C) treatment in AML cells is partially dependent on CDK activity. (**a** and **b**) OCI-AML3 (panel a) and THP-1 (panel b) cells were treated with cytarabine and AZ20, alone or in combination, for 24 h or 48 h. Whole cell lysates were subjected to Western blotting and probed with the indicated antibodies. Densitometry measurements normalized to β-actin and then compared to control are presented. Western blots were repeated at least three times and one representative cropped blot is shown. (**c** and **d**) OCI-AML3 and THP-1 cells were treated with cytarabine and AZ20, alone or in combination, for 24 h or 48 h. Then the cells were fixed with ethanol and stained with PI for cell cycle analysis.

To begin to investigate the molecular mechanism underlying the synergistic antileukemic interactions between AZ20 and cytarabine in AML cells, we treated AML cell lines with both drugs alone or in combination and determined the effects on CDK1. Cytarabine treatment caused increase of p-CDK1, while AZ20 treatment caused decrease of p-CDK1, which was further decreased following combined AZ20 and cytarabine treatment (Fig. 6a,b). Then we looked at the effects of AZ20 and cytarabine on cell cycle progression, alone or in combination. Cytarabine treatment led to S and G2/M arrest, which was abrogated by the addition of AZ20 (Fig. 6c,d). Similar results were obtained for AZD6738 in combination with cytarabine in OCI-AML3 cells (Fig. S1c,d).

**Figure 7:**
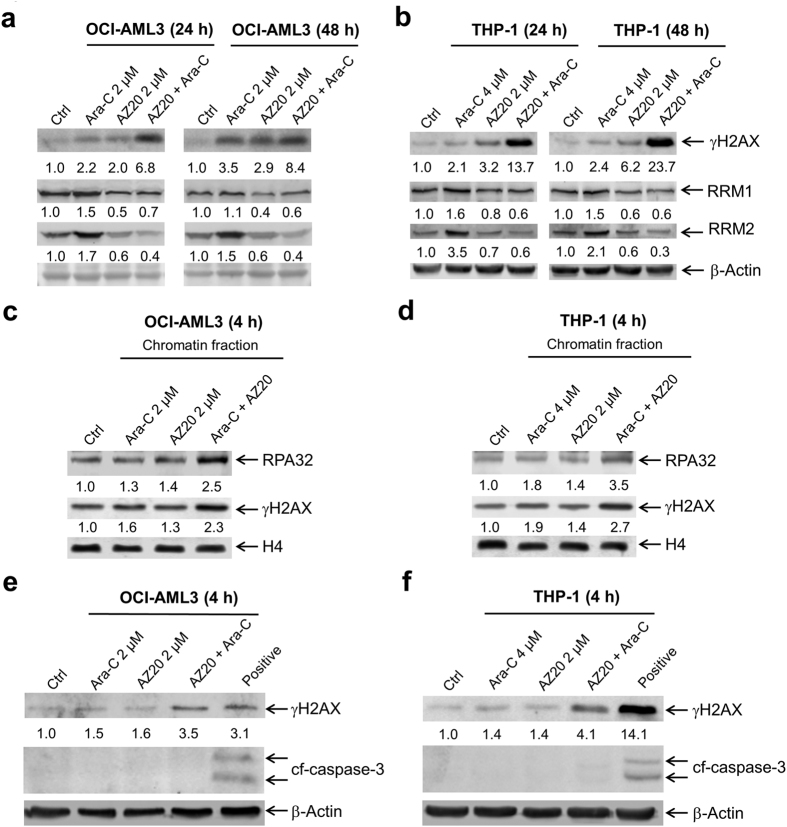
Combined AZ20 and cytarabine (ara-C) treatment causes enhanced DNA replication stress and damage, followed by apoptosis. (**a** and **b**) OCI-AML3 (panel a) and THP-1 (panel b) cells were treated with cytarabine and AZ20, alone or in combination, for 24 h or 48 h. Whole cell lysates were subjected to Western blotting and probed with the indicated antibodies. Western blots were repeated at least three times and one representative cropped blot is shown. (**c** and **d**) OCI-AML3 (panel c) and THP-1 (panel d) cells were treated with cytarabine and AZ20, alone or in combination, for 4 h. Chromatin-bound RPA32 and γH2AX were analyzed by Western blotting. Western blots were repeated at least three times and one representative cropped blot is shown. (**e** and **f**) OCI-AML3 and THP-1 cells were treated with cytarabine and AZ20, alone or in combination, for 4 h. Whole cell lysates were subjected to Western blotting and probed with the indicated antibodies. Whole cell lysates from AML cells treated with combined cytarabine and AZ20 for 48 h were used as the positive controls. Densitometry measurements normalized to β-actin or histone H4 and then compared to control are presented in panels a–f. Western blots were repeated at least three times and one representative cropped blot is shown.

We next looked at DNA damage induced by the combined drug treatment. As expected, cytarabine treatment caused increased expression of γH2AX in both OCI-AML3 and THP-1 cell lines, which was further increased by the addition of AZ20, indicating enhanced DNA damage induced by the combined treatment (Fig. 7a,b). Interestingly, cytarabine treatment also caused increased expression of both RRM1 and RRM2 in the cells, which was completely abolished by AZ20 (Fig. 7a,b). Similar results were obtained in OCI-AML3 cells treated with AZD6738 in combination with cytarabine (Fig. S1e). To confirm that the combined treatment indeed caused increased DNA damage, the AML cell lines were treated for a shorter time, 4 h, and then cellular fractionation was performed. There was an increase of chromatin-bound RPA32 in the combined treatment compared to individual treatments (Fig. 7c,d). Enhancement of chromatin-bound γH2AX by AZ20 was also detected 4 h following the combined treatment. Western blot analysis of whole cell lysates showed enhanced γH2AX in the combined drug treatment compared to individual treatments without detectable cleavage of caspase-3, providing evidence that the increased γH2AX was due to DNA damage and not apoptosis-induced DNA fragmentation (Fig. 7e,f). Essentially the same results were obtained in OCI-AML3 cells treated with AZD6738 in combination with cytarabine (Fig. S1f). These results demonstrate that combined cytarabine and AZ20 or AZD6738 treatment caused increased ssDNA and DNA damage, prior to induction of apoptosis.

## Discussion

Unacceptably low overall survival rates for AML patients have led to the realization that new therapies or rationally designed combination therapies are needed to improve treatment outcomes for AML patients. ATR plays a key role in the DNA damage response and has been identified as a potential therapeutic target in combination with DNA damaging agents^7^,^9^. Tibes and colleagues performed a kinome-wide screen to determine cytarabine sensitizers in AML cells and identified ATR, among others, as a cytarabine sensitizer^16^. ATR plays a key role in multiple cellular functions, including but not limited to: cell-cycle checkpoints, inhibition of replication origin firing, protection of stressed replication forks, and DNA repair^7^. While ATR inhibitors have been investigated in combination with DNA damaging agents, the mechanism of action is not fully understood.

In this study, we examined the mechanism of action of ATR inhibition by using selective ATR inhibitor AZ20 or AZD6738, alone and in combination with cytarabine in AML cells. We found that ATR inhibition caused downregulation of RRM2. Although inhibition of ATR has been shown to decrease the expression of RRM2 via CHK1 and E2F1 in a CDK-dependent manner^15^,^17^, we did not detect a change in the protein levels for E2F1 and found that downregulation of RRM2 was CDK1-independent. Our results suggest that in response to ATR inhibition a CDK1-independent mechanism of downregulation of RRM2 exists in AML cells. A surprising finding from this study was that ATR inhibition also caused downregulation of RRM1. We speculate that since RRM1 is generally steady throughout the cell cycle and it has a long half-life of about 15 h^18^,^19^, that the downregulation was likely due to changes on protein stability because decreased levels were detected as early as 4 h after treatment. Studies are underway to investigate how ATR inhibition downregulates RRM1 and RRM2 in AML cells. Interestingly, ATR inhibition caused increased DNA replication stress and DNA damage, accompanied by downregulation of RRM1, RRM2, and p-CDK1 (Y15). Finally, 8 h post-treatment, increased apoptosis was detected. Taken together, our results provide evidence to suggest that in AML cells, inhibition of ATR induces DNA replication stress, downregulation of RRM1 and RRM2 (resulting in further DNA replication stress), abrogation of the G2/M cell cycle checkpoint, and increased DNA damage, leading to induction of apoptosis.

Similar to studies using other DNA damaging agents in combination with ATR inhibitors, we found that inhibition of ATR synergized with cytarabine to induce apoptosis in AML cells. Combination of ATR inhibition and cytarabine treatment resulted in increased DNA replication stress and DNA damage, prior to detection of cleaved caspase-3, indicating that they occurred before apoptosis. Cytarabine treatment resulted in upregulation of both RRM1 and RRM2, which was completely abrogated by combined treatment with the ATR inhibitor AZ20 or AZD6738. ATR inhibition prevented cytarabine-induced cell cycle arrest, suggesting that apoptosis induced by the combined treatment was at least partially dependent on abrogation of the cell cycle checkpoints.

In summary, our results provide insight into the mechanism of action for the synergistic antileukemic activity of the ATR inhibitor AZ20 or AZD6738 in combination with cytarabine in AML cells. Our results provide evidence that induction of DNA replication stress and DNA damage, and abrogation of the cell cycle checkpoints contribute to the synergistic antileukemic activity of ATR inhibition in combination with cytarabine treatment. Though, further confirmation of the mechanism of action in more AML cell lines and patient samples of varying genetic backgrounds is warranted. In addition, other mechanisms may also contribute to the antileukemic activity of the combination treatment. Our study supports the further development of ATR inhibitors in combination with cytarabine for the treatment of AML.

## Materials and Methods

### Drugs

AZ20, AZD6738, and RO-3306 were purchased from Selleck Chemicals (Houston, TX, USA). Cytarabine was purchased from Sigma-Aldrich (St. Louis, MO, USA).

### Cell Culture

THP-1 and MV4-11 cell lines were purchased from the American Type Culture Collection (Manassas, VA, USA). The CTS cell line was a gift from Dr. A Fuse from the National Institute of Infectious Diseases, Tokyo, Japan. The OCI-AML3 cell line was purchased from the German Collection of Microorganisms and Cell Cultures (DSMZ, Braunschweig, Germany). MOLM-13 cells were purchased from AddexBio (San Diego, CA, USA). The cell lines were cultured in RPMI 1640 (except OCI-AML3, which was cultured in alpha-MEM) with 10–15% fetal bovine serum (Life Technologies, Grand Island, NY, USA), 2 mM L-glutamine, 100 U/ml penicillin and 100 μg/ml streptomycin. All the AML cell lines were cultured in a 37 °C humidified atmosphere containing 5% CO2/95% air and tested for the presence of mycoplasma on a monthly basis.

Diagnostic AML blast samples derived from patients either at initial diagnosis or at relapse were purified by standard Ficoll-Hypaque density centrifugation, then cultured in RPMI 1640 with 20% fetal bovine serum supplemented with ITS solution (Sigma-Aldrich) and 20% supernatant of the 5637 bladder cancer cell line (as a source of granulocyte-macrophage colony-stimulating factor)^20^,^21^,^22^,^23^,^24^.

### Clinical Samples

Diagnostic AML blast samples were obtained from the First Hospital of Jilin University, Changchun, China. Written informed consent was provided according to the Declaration of Helsinki. This study was approved and carried out in accordance with the guidelines set forth by the Human Ethics Committee of the First Hospital of Jilin University. Clinical samples were screened for gene mutations by PCR amplification and automated DNA sequencing and for fusion genes by real-time RT-PCR, as described previously^20^,^25^. Patient characteristics are shown in Table 1.

### *In Vitro* Cytotoxicity Assays

*In vitro* cytotoxicities of AZ20 and cytarabine, alone or combined, in AML cells were measured by using MTT (3-[4,5-dimethyl-thiazol-2-yl]-2,5-diphenyltetrazoliumbromide, Sigma-Aldrich), as previously described^26^,^27^. Briefly, 50 μl of cells, at a density of 2–5 × 10^5 ^cells/mL for cell lines and 50,000 cells/well at a density of 1 × 10^6^ cells/mL for patient samples were treated with variable concentrations of AZ20 and cytarabine, alone or in combination, for 72 hours. MTT was added to a final concentration of 1 mM and cells were incubated for 4 hours at 37 °C. The cells were lysed overnight using 10% SDS in 10 mM HCl and plates were read at 590 nm using a microplate reader. IC_50_ values were calculated as drug concentrations necessary to inhibit 50% growth compared to vehicle control treated cells. The IC_50_ values for the patient samples are means of duplicates from one experiment, due to limited sample. Patient samples for the combined drug treatments were chosen solely based on sample availability. The extent and direction of the antileukemic interactions between cytarabine and AZ20 were determined by standard isobologram analyses, as previously described^26^,^28^,^29^.

### Western Blot Analysis

Cell lines were harvested in log-phase growth, seeded at a density of 3 × 10^5 ^cells/mL (THP-1) or 5 × 10^5 ^cells/mL (OCI-AML3) and incubated with the indicated drugs for up to 48 h, as indicated. Cells were lysed in the presence of protease and phosphatase inhibitors (Roche Diagnostics, Indianapolis, IN, USA). Whole cell lysates were subjected to SDS-polyacrylamide gel electrophoresis, electrophoretically transferred onto polyvinylidene difluoride (PVDF) membranes (Thermo Fisher Inc., Rockford, IL, USA) and immunoblotted as previously described^24^,^26^,^30^,^31^. Immunoreactive proteins were visualized using the Odyssey Infrared Imaging System (Li-Cor, Lincoln, NE, USA), as described by the manufacturer. Western blots were repeated at least three times and one representative cropped blot is shown.

### Apoptosis

One million AML cells (3 × 10^5 ^cells/mL for THP-1, MOLM13, CTS and MV4-11, 5 × 10^5 ^cells/mL for OCI-AML3, or 1 × 10^6 ^cells/mL for patient samples) were treated with the indicated drugs, alone or in combination, for 24 or 48 h, and then subjected to flow cytometry analysis to determine drug-induced apoptosis using an Annexin V-fluorescein isothiocyanate (FITC)/ PI apoptosis Kit (Beckman Coulter; Brea, CA, USA), as previously described^26^,^32^. Experiments with AML cell lines were performed 3 independent times in triplicates, while patient sample experiments were performed once in triplicate due to limited sample. Data are presented as mean ± standard errors from one representative experiment. Patient samples were chosen based on availability of adequate sample for the assay. The extent and direction of antileukemic interactions between the two drugs were determined by calculating the combination index (CI) values using CompuSyn software (Combosyn Inc., Paramus, NJ, USA). CI < 1, CI = 1, and CI > 1 indicate synergistic, additive, and antagonistic effects, respectively^26^,^29^.

### Cell Cycle Progression

One million AML cells (3 × 10^5 ^cells/mL for THP-1 or 5 × 10^5 ^cells/mL for OCI-AML3) were treated with the indicated drugs for up to 48 h. The cells were harvested and fixed with ice-cold 80% (v/v) ethanol for 24 h. The cells were pelleted, washed with PBS, and resuspended in PBS containing 50 μg/mL PI, 0.1% Triton X-100 (v/v), and 1 μg/mL DNase-free RNase. DNA content was determined by flow cytometry analysis using a FACS Calibur flow cytometer (Becton Dickinson, San Jose, CA, USA), as previously described^28^. Cell cycle analysis was performed using Multicycle software (Phoenix Flow Systems, Inc., San Diego, CA, USA). Histograms were created using FlowJo v7.6.5 (Tree Star, Ashland, OR, USA). Cell cycle experiments were performed 3 independent times; histograms from one representative experiment are shown.

### Chromatin Fractionation

AML cell lines (3 × 10^5 ^cells/mL for THP-1 or 5 × 10^5 ^cells/mL for OCI-AML3) were treated with the indicated drugs for up to 24 h. Chromatin fractionation was carried out as described by Buisson and colleagues^15^. 3 × 10^6^ cells were washed with PBS and resuspended in solution A (10 mM HEPES, pH 7.9, 10 mM KCl, 1.5 mM MgCl_2_, 0.34 M sucrose, 10% glycerol, 1 mM DTT, 10 mM NaF, 1 mM Na_2_VO_3_ and protease inhibitors). Triton X-100 was added (final concentration of 0.1%) and then the cells were incubated on ice for 5 min. Nuclei were separated from cytoplasmic proteins by centrifugation at 1400× g for 4 min and then washed with solution A three times. Nuclei were then lysed in 3 mM EDTA, 0.2 mM EGTA, 1 mM DTT and protease inhibitors (dissolved in water) for 30 min at 4 °C. Chromatin was separated from soluble nuclear proteins by centrifugation at 1700 × g for 4 min. Soluble nuclear proteins were combined with cytoplasmic proteins (designated soluble fraction). Chromatin was washed three times with nuclei lysis buffer (centrifugation was carried out at 1700 × g for 4 min). Chromatin was resuspended in 200 μl Laemmli sample buffer and sonicated. These experiments were repeated three independent times and blots from one representative experiment are shown.

### Statistical Analysis

Differences in cell apoptosis between treated (individually or combined) and untreated cells were compared using the pair-wise two-sample t-test or repeated measures 1-way ANOVA with Bonferroni post hoc test. Differences in AZ20 IC_50_s between t(8;21) and t(15;17) vs. all other samples was calculated using the Mann-Whitney *U*-test. Statistical analyses were performed with GraphPad Prism 5.0. Error bars represent ± SEM. The level of significance was set at *p* < 0.05.

## Additional Information

**How to cite this article**: Ma, J. *et al*. Mechanisms responsible for the synergistic antileukemic interactions between ATR inhibition and cytarabine in acute myeloid leukemia cells. *Sci. Rep.* **7**, 41950; doi: 10.1038/srep41950 (2017).

**Publisher's note:** Springer Nature remains neutral with regard to jurisdictional claims in published maps and institutional affiliations.

## Supplementary Material

Supplemental Figure S1

## Figures and Tables

**Table 1:** Patient characteristics of primary AML patient samples.

Patient	Gender	Age (year)	Disease status	Cytogenetics	Gene mutation	Blast purity (%)
AML#31	Male	17	Newly diagnosed	46, XY	CEBPA double mutation	68.5
AML#33	Female	76	Newly diagnosed	46, XX	dupMLL, CEBPA mutation	84.5
AML#34	Male	52	Newly diagnosed	46, XY	DEK/CAN	96
AML#35	Male	65	Newly diagnosed	47, XY, add(7q), -16, -17, +marx3		76
AML#36	Male	43	Newly diagnosed	46, XY, t(8;21)(q22;q22)	AML1-ETO	48
AML#39	Male	50	Newly diagnosed	45, X, -Y, t(8;21)(q22;q22), del(11q)	AML1-ETO	46
AML#40	Male	12	Newly diagnosed	46, XY, t(15;17)(q22;q21)	PML-RARα	92.5
AML#41	Male	74	Newly diagnosed	47, XY, +8	FLT-3 ITD, NPM-1 and DNMT3A mutation	95
AML#43	Male	19	Newly diagnosed	45, X, -Y, t(8;21)(q22;q22), del(9q)	AML1-ETO	47
AML#44	Male	25	Newly diagnosed	46, XY, t(15;17)(q22;q21)	PML-RARα	94
AML#45	Male	48	Relapsed	46, XY, t(7;11)(p15;p15)	FLT-3 ITD	39.5
AML#46	Female	9	Newly diagnosed	NA	NA	93.5
AML#47	Female	50	Relapsed	46, XX	CEBPA double mutation	81
AML#48	Female	7	Newly diagnosed	46, XX, t(11;20)(p15;q11)/46, idem, del(9)(q22)		83
AML#49	Female	52	Newly diagnosed	46, XX, t(15;17)(q22;q21)	PML-RARα	90
AML#50	Male	38	Newly diagnosed	47, XY, add(1p), t(15;17)(q22;q21), +14	PML-RARα	95
AML#51	Male	34	Newly diagnosed	46, XY	FLT-3 ITD, dupMLL	29
AML#52	Female	51	Newly diagnosed	46, XX		82
AML#53	Male	48	Newly diagnosed	46, XY	IDH2 and DNMT3A mutation	42
AML#55	Female	77	Newly diagnosed	46, XY		50
AML#56	Female	44	Newly diagnosed	46, XX, t(15;17)(q22;q21)	PML-RARα	89
AML#57	Female	12	Newly diagnosed	47, XX, +10	FLT-3 ITD, CEBPα mutation	80
AML#58	Female	60	Newly diagnosed	46, XX		69.5
AML#59	Female	32	Newly diagnosed	46, XX, del(9q)	CEBPA double mutation	27
AML#60	Female	65	Newly diagnosed	46, XX	FLT-3 ITD, NPM-1 mutation	91
AML#61	Male	18	Newly diagnosed	46, XY, t(15;17)(q22;q21)	PML-RARα	95
AML#62	Male	64	Newly diagnosed	46, XY		69
AML#63	Male	59	Newly diagnosed	46, XY	HOX11 positive	82
AML#64	Female	75	Newly diagnosed	46, XX, +8		91
AML#65	Female	54	Newly diagnosed	46, XX	MLL-AF6	64
AML#66	Female	48	Newly diagnosed	45, XX. del(3q), −7		39
AML#92	Male	64	Relapsed	46, XY		85
