# Supplementary material for: Mechanisms responsible for the synergistic antileukemic interactions between ATR inhibition and cytarabine in acute myeloid leukemia cells
Source: Sci Rep. 2017 Feb 8;7:41950. doi: 10.1038/srep41950 (PMC5296912; doi:10.1038/srep41950)
Supplement: Supplemental Figure S1 [file srep41950-s1.pdf]

Mechanisms responsible for the synergistic antileukemic interactions between ATR inhibition and cytarabine in acute myeloid leukemia cells

Jun Ma[1], Xinyu Li[1], Yongwei Su[1], Jianyun Zhao[1,2], Daniel A. Luedtke[3], Valeria Epshteyn[2], Holly Edwards[4,5], Guan Wang[1], Zhihong Wang[2,6], Roland Chu[2,6], Jeffrey W. Taub[2,6], Hai Lin[7], Yue Wang[8,*], and Yubin Ge[2,3,4,5*]

[1] National Engineering Laboratory for AIDS Vaccine, Key Laboratory for Molecular Enzymology and Engineering, the Ministry of Education, School of Life Sciences, Jilin University, Changchun, P. R. China
[2] Department of Pediatrics, Wayne State University School of Medicine, Detroit, MI, USA
[3] Cancer Biology Graduate Program, Wayne State University School of Medicine, Detroit, MI
[4] Department of Oncology, Wayne State University School of Medicine, Detroit, MI, USA
[5] Molecular Therapeutics Program, Barbara Ann Karmanos Cancer Institute, Wayne State University School of Medicine, Detroit, MI, USA
[6] Division of Pediatric Hematology/Oncology, Children's Hospital of Michigan, Detroit, MI, USA
[7] Department of Hematology and Oncology, The First Hospital of Jilin University, Changchun, P. R. China
[8] Department of Pediatric Hematology and Oncology, The First Hospital of Jilin University, Changchun, P. R. China

* Corresponding author

Address correspondence and reprint requests:
Yubin Ge, Ph.D.
Departments of Oncology and Pediatrics
Wayne State University School of Medicine
110 East Warren Ave.
Detroit, Michigan 48201, USA
Tel: (313) 578-4285
Fax: (313) 578-4287
Email: gey@karmanos.org

Or

Yue Wang, M.D. and Ph.D.
Department of Pediatric Hematology and Oncology
The First Hospital of Jilin University
Changchun, P. R. China
Tel: 011-86-431-88782970
Email: wangyue7373@126.com

Figure S1

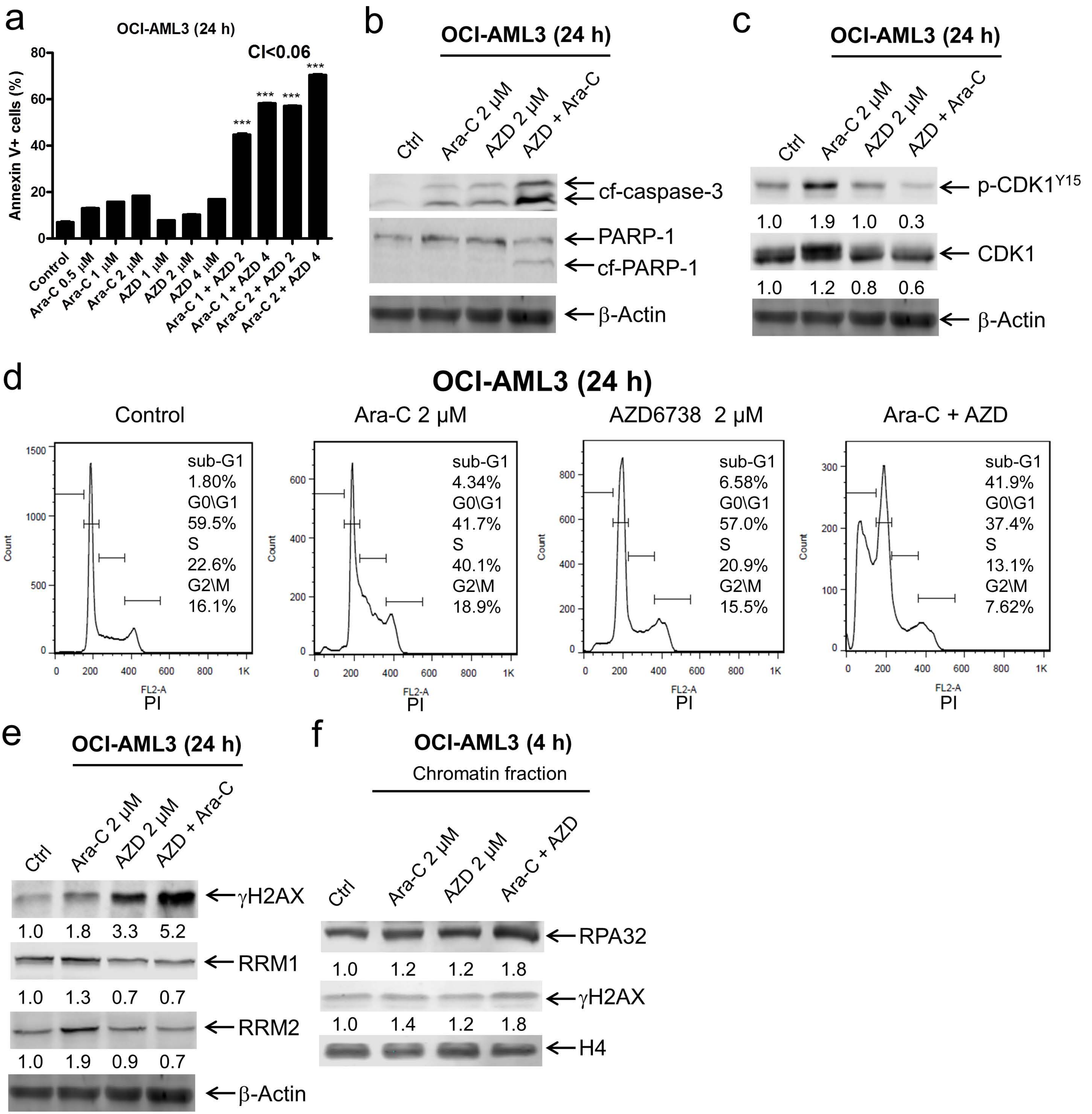

a
OCI-AML3 (24 h)
CI<0.06
Annexin V+ cells (%)
80
60
40
20
0
***
Control
Ara-C 0.5 μM
Ara-C 1 μM
Ara-C 2 μM
AZD 1 μM
AZD 2 μM
AZD 4 μM
Ara-C 1 + AZD 2
Ara-C 1 + AZD 4
Ara-C 2 + AZD 2
Ara-C 2 + AZD 4
b
OCI-AML3 (24 h)
Ctrl
Ara-C 2 μM
AZD 2 μM
AZD + Ara-C
cf-caspase-3
PARP-1
cf-PARP-1
β-Actin
c
OCI-AML3 (24 h)
Ctrl
Ara-C 2 μM
AZD 2 μM
AZD + Ara-C
p-CDK1Y15
1.0 1.9 1.0 0.3
CDK1
1.0 1.2 0.8 0.6
β-Actin
d
OCI-AML3 (24 h)
Control
sub-G1 1.80%
G0\G1 59.5%
S 22.6%
G2\M 16.1%
Ara-C 2 μM
sub-G1 4.34%
G0\G1 41.7%
S 40.1%
G2\M 18.9%
AZD6738 2 μM
sub-G1 6.58%
G0\G1 57.0%
S 20.9%
G2\M 15.5%
Ara-C + AZD
sub-G1 41.9%
G0\G1 37.4%
S 13.1%
G2\M 7.62%
Count
FL2-A
PI
e
OCI-AML3 (24 h)
Ctrl
Ara-C 2 μM
AZD 2 μM
AZD + Ara-C
γH2AX
1.0 1.8 3.3 5.2
RRM1
1.0 1.3 0.7 0.7
RRM2
1.0 1.9 0.9 0.7
β-Actin
f
OCI-AML3 (4 h)
Chromatin fraction
Ctrl
Ara-C 2 μM
AZD 2 μM
Ara-C + AZD
RPA32
1.0 1.2 1.2 1.8
γH2AX
1.0 1.4 1.2 1.8
H4

Figure S1. AZD6738 (AZD) synergizes with cytarabine (ara-C) to induce apoptosis and proliferation inhibition in AML cells. (a) OCI-AML3 cells were treated with cytarabine and AZD6738, alone or in combination, for 24 h and then subjected to annexin V-FITC/PI staining and flow cytometry analyses. CI values were calculated using CompuSyn software. Combined drug treatments were compared to single drug treatment using 1-way ANOVA with Bonferroni post hoc test. ***indicates $p < 0.001$. (b and c) OCI-AML3 cells were treated with cytarabine and AZD-6738, alone or in combination, for 24 h. Whole cell lysates were subjected to Western blotting and probed with the indicated antibodies. (d) OCI-AML3 cells were treated with cytarabine and AZ20, alone or in combination, for 24 h. Then the cells were fixed with ethanol and stained with PI for cell cycle analysis. (e) OCI-AML3 cells were treated with cytarabine and AZD6738, alone or in combination, for 24 h. Whole cell lysates were subjected to Western blotting and probed with the indicated antibodies. (f) OCI-AML3 cells were treated with cytarabine and AZ20, alone or in combination, for 4 h. Chromatin-bound RPA32 and γH2AX were analyzed by Western blotting. Densitometry measurements normalized to β-actin or histone H4 and then compared to control are presented.
